# Knowledge Reasoning with Semantic Data for Real-Time Data Processing in Smart Factory

**DOI:** 10.3390/s18020471

**Published:** 2018-02-06

**Authors:** Shiyong Wang, Jiafu Wan, Di Li, Chengliang Liu

**Affiliations:** 1 School of Mechanical & Automotive Engineering, South China University of Technology, Guangzhou 510640, China; mesywang@scut.edu.cn (S.W.); itdili@scut.edu.cn (D.L.); 2 School of Mechanical Engineering, Shanghai Jiao Tong University, Shanghai 200240, China; chlliu@sjtu.edu.cn

**Keywords:** smart factory, knowledge reasoning, data analysis, ontology, semantic data

## Abstract

The application of high-bandwidth networks and cloud computing in manufacturing systems will be followed by mass data. Industrial data analysis plays important roles in condition monitoring, performance optimization, flexibility, and transparency of the manufacturing system. However, the currently existing architectures are mainly for offline data analysis, not suitable for real-time data processing. In this paper, we first define the smart factory as a cloud-assisted and self-organized manufacturing system in which physical entities such as machines, conveyors, and products organize production through intelligent negotiation and the cloud supervises this self-organized process for fault detection and troubleshooting based on data analysis. Then, we propose a scheme to integrate knowledge reasoning and semantic data where the reasoning engine processes the ontology model with real time semantic data coming from the production process. Based on these ideas, we build a benchmarking system for smart candy packing application that supports direct consumer customization and flexible hybrid production, and the data are collected and processed in real time for fault diagnosis and statistical analysis.

## 1. Introduction

Smart factories and smart production are drawing increasing attention from both the academic community and industrial pioneers [1]. The smart factory aims to construct manufacturing-oriented cyber-physical systems to implement vertical integration of automation and information to finally achieve digitization and intelligence [2]. Within a smart factory, automated machines and the cloud are connected via networks, enabling the collection of mass data and the deployment of various kinds of software [3,4,5]. Industrial big data analytics is believed to be a promising and indispensable enabler for the implementation of smart factories. Based on data analysis, physical objects are likely to be operated, monitored, and maintained in a rather smart way, exhibiting flexibility, efficiency, and transparency [6,7].

Big data analytics plays an essential role in a smart factory. It acts like a brain, extracting valuable knowledge to serve decision-making in different layers to support applications such as global performance optimization, supervisory control, and fault diagnosis, as well as proactive operation and maintenance [8,9]. There are related algorithms and architectures that are already available and developing rapidly. The Google Map-Reduce architecture and its open-source version of Hadoop enable a cluster of thousands of computers to work together to store and process mass data. Based on the Deep Learning algorithm, a novel machine-learning method, the AI application called AlphaGo invented by the DeepMind company defeated some world champions at Go. Therefore, Deep Learning-like algorithms executed by Hadoop-like distributed architectures running on the cloud could be a promising methodology for revealing the inner logic of data.

Big data arose from Internet applications, where its characteristics have been summarized as the 5 Vs, i.e., Volume, Velocity, Variety, Value, and Veracity, which mean information being generated at a high volume (e.g., terabytes per day), with a rapid rate of change, encompassing a broad range of sources including both structured and unstructured data, containing valuable information, and related to the real world. However, unstructured data, wrong information, and low value density may cause inefficiency and ineffectiveness when processing Internet big data. On the contrary, considering that the industrial production is a precisely controlled process, except for the rapidly increasing volume, industrial data can be well-defined with rich semantics, fine structures, and guaranteed correctness and accuracy, which may help simplify data analysis. On the other hand, most industrial data need real-time and online processing to serve process monitoring and dynamical decision-making, which makes offline analysis unsuitable.

To address the real-time processing requirements of increasing industrial data, we introduce the knowledge reasoning method. Our contributions are mainly focused on two aspects. First, we present a framework for smart factories to arrange involved components into a layered and structural architecture, based on which we could identify different data processing hosts and separating online real-time processing from offline batch processing. From top to bottom, client terminals, cloud, and shop-floor entities are organized into two loops. On the one hand, the framework couples the self-organization of shop-floor entitles with the global coordination of cloud. During self-organization, products, machines, and conveyors negotiate with each other to organize production. The cloud helps to solve global problems such as deadlock prevention and performance optimization. On the other hand, the cloud supports client terminals for consumer ordering, information access, and visualization. Second, we propose a scheme considering the generation of semantic data, the integration of the semantic data with the ontology model, and the operation of the ontology model with a reasoning engine. Finally, we use a smart factory prototype, simulating a personalized candy packing application, to verify the proposed architecture and method.

The article is organized as follows. The framework for the smart factory is presented in Section 2. Then the knowledge reasoning based on an online real-time data processing method is proposed in Section 3. To verify the framework and method, we build a smart factory prototype for packing assorted candies serving personalized consumption in Section 4. Finally, the paper is concluded in Section 5.

## 2. Framework of the Smart Factory

Physical and cyber components cooperate together in smart factories, and they function in different layers and play different roles. In this section, we present a framework to reveal the organizational structure of the smart factory components. After that, we analyze the computing ability and data processing burden of the smart factory components laying a foundation for the later data-processing scheme.

### 2.1. Organizational Structure of Smart Factory Components

**Figure 1:**
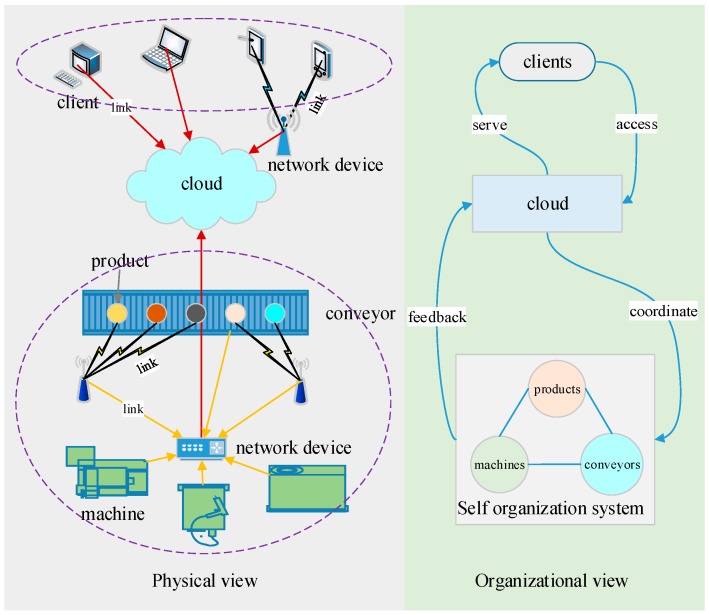
Framework for organizing production-related hardware components of a smart factory.

From a hardware point of view, machines (e.g., CNC machine tools), conveyors (e.g., conveyor belts, AGVs, and robotic arms), products (e.g., raw materials, parts, and components), cloud (public or private, providing virtual machines), client terminals (e.g., computers and smart cell phones), and network (general/industrial networks in the wired/wireless form) are directly related to production. Figure 1 describes the roles and functions of these components. The machines process products and the conveyors transport products among machines. The cloud, public or private, provides flexible computing ability and storage room for data and software. Client terminals serve as interfaces between people and the production system in different levels. Local terminals generally refer to the GUIs of devices. Remote terminals are independent of machines as they serve for system-level monitoring. The networks interconnect machines, conveyors, and products on the one hand, and on the other hand, connect these physical entities and the client terminals to the cloud.

When a production task comes down to the smart factory, machines, conveyors, and products negotiate with each other to organize resources for completing the task. A product knows which operations it needs, such that it calls for the machines to bid for the operations and the conveyors to delivery it among the winning machines. This kind of negotiation features self-organization, fault tolerance, and high dynamics. First, self-organization means machines, conveyors, and products are peer partners; in other words, no central commander exists. Second, the high dynamics comes from the intelligent negotiation that occurs from one operation to another; during negotiation, the resources are determined in real time instead of based on pre-allocation. Third, the highly dynamical self-organization results in fault tolerance, as faulty machines or conveyors can be excluded easily and in time, because they will simply ignore the bidding invitation. During production, the cloud will participate in the negotiation in order to optimize global performance. However, the participation of the cloud is not necessary so that when the cloud malfunctions the production can continue, which is another contribution to fault tolerance. Therefore, the production operates in a self-organized way with assistance of the cloud.

### 2.2. Analysis of Computing Ability and Data Processing Burden of the Smart Factory Components

**Figure 2:**
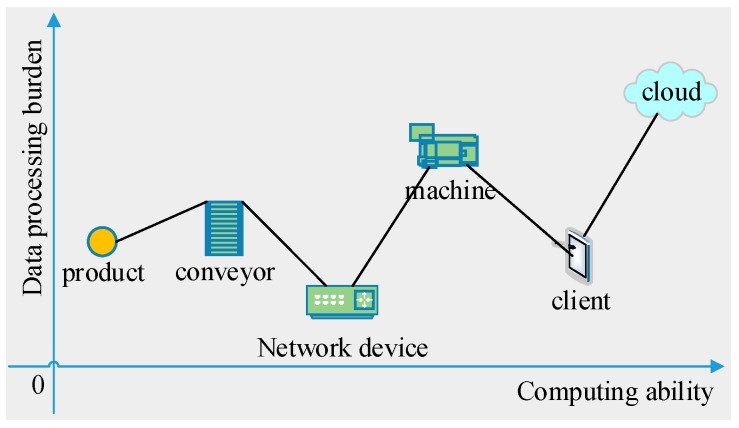
Relative computing ability and data processing burden of the smart factory components.

As shown in Figure 2, the cloud, client terminals, and network devices are computer-like equipment so as to be able to compute and store data. The machines, conveyors, products are also armed with computerized controllers. Therefore, all these components have some computing ability and storage capacity. The cloud has the most powerful computing resource to accommodate data and software; the client terminals are mainly used to display graphs or GUIs, and little analysis needs doing in the terminals; network devices are for communication, so that user reprograming is not needed. While the products do not need to be controlled by physical actions, the conveyors need simple control and the machines need complex control. Therefore, the machines and the cloud are two main components responsible for the industrial data analysis.

To facilitate both self-organization and cloud assistance, separate network technologies should be used. An independent private network is required for the self-organization process. The guaranteed information security and QoS are more easily achieved with a private network. Through a gateway, the self-organized network can be linked to the cloud through another network that should suit for mass data transfer and security. The Internet is currently an ideal solution to connect terminals to the cloud. With the network, the cloud, and the smart processors, data can be generated, interchanged, and processed along with the production process.

## 3. Integration of Semantic Data and Knowledge Reasoning

**Figure 3:**
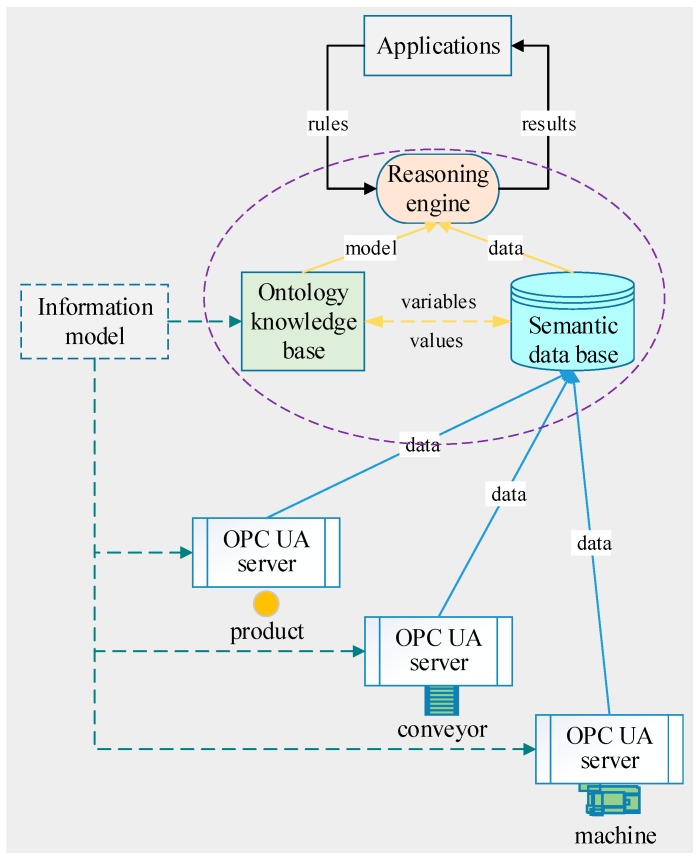
Integrated architecture of knowledge reasoning and semantic data.

Primitive data introduces difficulties during data analysis especially when the volume of data significantly increases. Therefore, we need semantic data to conduct intelligent analysis and application. Figure 3 describes a combination of ontology-based knowledge modeling, OPC UA-based sematic data generation, and semantic data base to implement real-time processing of industrial data. Information models for machines, conveyors, products, and the system should have been built previously. Based on the information model, the concepts in the domain and also the relationships that hold between those concepts can be defined with a software tool, like Protégé, to create an ontology model. According to the same information model, OPC UA address spaces are created for the machines, conveyors, and products, and the ontology is created for the system.

The overall data processing works like this. The OPC UA servers, the semantic data base, and the reasoning engine operate concurrently, but interact with one another through data. Each product, machine, and conveyor collects its data and stores it in its OPC UA address space. These data will be duplicated and loaded up into the semantic data base on the cloud. The reasoning engine will process the ontology model periodically. At the beginning of reasoning, the data stored in the data base will be assigned to the datatype properties of the ontology objects, and the rules coming from the upper applications will be applied. After reasoning, results will be feedback to application and the decision-making agent. The use of mature reasoning engines along with definable rules instead of programming contributes flexibility, stability, and simplicity.

### 3.1. Information Modeling

OPC UA expresses information in a similar way to ontology, where objects and references (describing the relationship between objects) are commonly used. Therefore, developing an independent information model in an object-oriented way is necessary. However, any object can be viewed as a system that consists of other objects. This means we cannot define every object that exists in our manufacturing system. One should focus on the application to simplify the information modeling. In our design, self-organization and cloud assistance are two core ideas where machines, conveyors, products organize themselves to process tasks, and the cloud assists this self-organized process to optimize system performance. Therefore, machines, conveyors, products are identified as the first level classes, which can be subdivided as in Table 1. Note that the classification of machines and conveyors based on functions instead of structures. For example, a robotic arm can be a processing machine if it is used for welding, but a conveyor if it is just used to move products from one place to another. Human beings are always an indispensable factor in production, and based on their duties, involved employee can be subdivided as well.

### 3.2. Software Tools for Implementation

The recommended modeling language and related software tools are summarized in Table 2. The UML (Unified Modeling Language) is a powerful and application independent modeling language to construct the information models. A number of software tools are available for UML modeling, and some of them are free and even open source, such as JUDE-community, Argo UML, UMLet, Visual paradigm-community, and BOUml. To help the development of OPC UA-compliant programs, a few software tools are already available in the market, among which the UaModeler from the Unified Automation Company is quite a popular one. Protégé is a respective tool for ontology modeling which supports OWL (Web Ontology Language) 2.0. Finally, Apache Jena is a free and open-source Java framework for building semantic web and linked data applications, supporting SWRL (Semantic Web Rule Language).

Therefore, we have a set of tools where the JUDE community uses UML to build the information model, based on which Protégé is used to build the ontology and the UaModeler is used to build the OPC UA model. The OPC UA servers provide semantic data to the cloud, combined with which the ontology is recurrently processed by the Jena, taking application requirements that are expressed with SWRL rules as input. The original ontology built by Protégé can be called a file model, and this model becomes a memory model after the file model is loaded into memory. After reasoning, the memory model turns into inference model. As the inference model reflects the change of the system, it may be different from the memory model. Fortunately, the inference model can be saved as a new file model so that the ontology model can evolve through reasoning.

### 3.3. Demonstration

**Figure 4:**
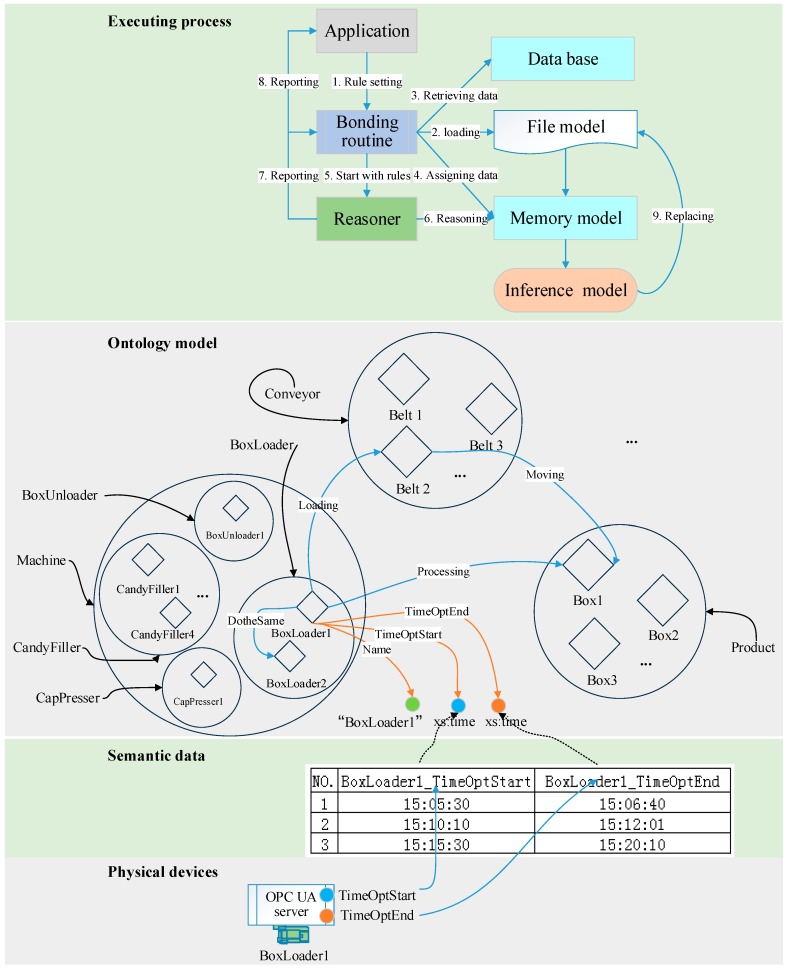
Illustration of integration of knowledge reasoning and semantic data.

In this section, we explore more details related to the integration of knowledge reasoning and semantic data using an imaginary and simple example as shown in Figure 4. With Protégé, we define three Classes called Product, Machine, and Conveyor. Each class has several individuals, i.e., instances or objects used by other technologies. For an individual, two types of properties can be defined, an object property linking the individuals (e.g., the property loading linking two individuals—BoxLoader1 to Belt2), and a datatype property actually defining variables for an individual [10]. Three datatype properties are defined for BoxLoader1 as an example. Name, TimeOptStart, and TimeOptEnd mean the name of the machine, the start time of a box loading operation, and the corresponding end time of the operation, respectively.

It is easy to see that the values for TimeOptStart, and TimeOptEnd cannot be determined in advance. They should be acquired from the shop floor production process. The BoxLoader1 in the psychical layer is responsible for recording these values into its OPC UA address space and sending them to the semantic data base that bridges the gap between the physical machines and the ontology model. This is because the properties of the ontology model correspond to the database fields, which in turn correspond to the OPC UA variables (Figure 4).

Figure 4 shows various components integrated through a Bonding routine. Prior to reasoning, a system user can set reasoning rules written in the SWRL language through applications (step 1). In this demonstration, a rule says, “greaterThan (TimeOptEnd,TimeOptStart+90), lessThan (TimeOptEnd,TimeOptStart+250)”, which means that the working time (i.e., the interval between operation end and operation start) should not be less than 90 s and should not be more than 250 s, either. In addition, the Bonding routine will load the ontology model from file to memory (step 2). Then, the Bonding routine retrieves values from the data base (step 3) and assigns them to objects of memory model (step 4). After that, the Bonding routine invokes the reasoner (step 5), Jena, to process the memory model (step 6) which will turn into an inference model. Checking against this rule, records No. 1 and No. 3, 70 s and 280 s for interval respectively, are exceptions, but record No. 2 is a normal situation, as its interval is 111 s. After reasoning, the reasoner reports the results to the Bonding routine (step 7), which in turn transfers the results to the application (step 8). The Bonding routine also saves the inference model to replace the previous file model to compete the model update (step 9). Then next execution is ready.

When the application receives the reasoning result about record no. 1, for example, it is told that something is wrong with the BoxLoader1, which uses less time than needed. The application will inform this exception to operators through client terminals, and it will also warn and stop BoxLoader1. Then, the application tries to solve this exception by asking the reasoner to find another machine to replace the BoxLoader1. The reasoner can find BoxLoader2, not by name but through the object property, DotheSame. Finally, the BoxLoader is activated and starts to participate in negotiation. This process is in accord with the proposed framework where the cloud coordinates the shop-floor entities and provides information to client terminals.

## 4. Personalized Candy Packing Benchmark

**Figure 5:**
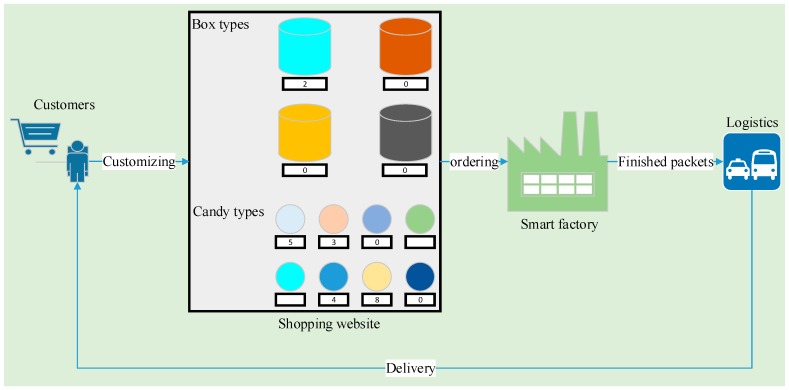
Integrated personalized candy packing prototype.

Based on the proposed framework and data processing scheme, we constructed a smart factory prototype. We present this prototype here as supporting material and for reference. If a consumer can select the box types, the candy types, and the number of candies of each type, they can customize their unique packet of candies, which can be used as an interesting gift. We address this kind of desire by providing an integrated solution, as shown in Figure 5. We developed a shopping website for consumers to customize their special packets of candies, to submit order, and to pay, similar to the well-known American Amazon website or Chinese Taobao website. The advantage of our UniqueCandy website is that the orders can be directly sent to the smart factory for automatic processing. This actually integrates the consumers with the smart factory to rapidly respond to consumer demanding.

**Figure 6:**
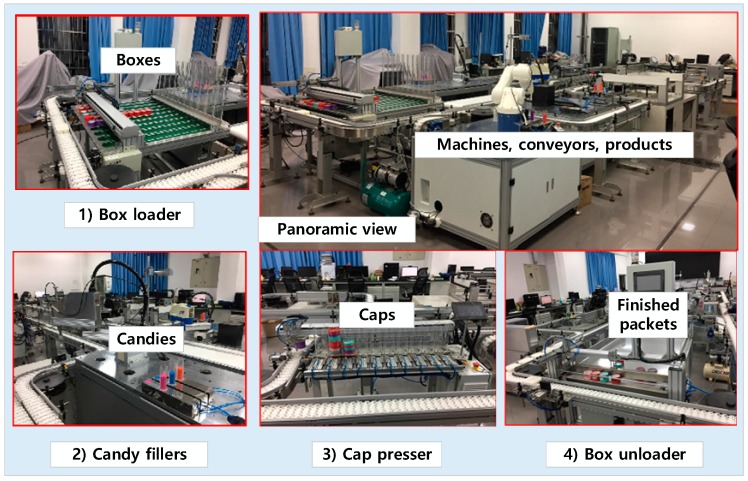
Snapshot of the smart factory called UniqueCandy.

The prototype smart factory, UniqueCandy, occupies about 150 square meters (Figure 6). There are three machines used to upload the boxes onto the conveyor belts, to cover the lids after all the candies required are put into the box, and to download the boxes off the conveyor belts after the lid covering operation. A RFID tag is attached on the surface of each box to store consumer customization information, i.e., the candy types and the number of candies of each type. In addition, during the locomotion of the box, it negotiates with candy loading machines and conveyor belts to determine machines and construct transportation routes. In the UniqueCandy benchmarking, five conveyor belts are deployed to build four intersecting loops featuring multiple branches. Four robotic arms are used as candy loading machines responsible for different candy types.

**Figure 7:**
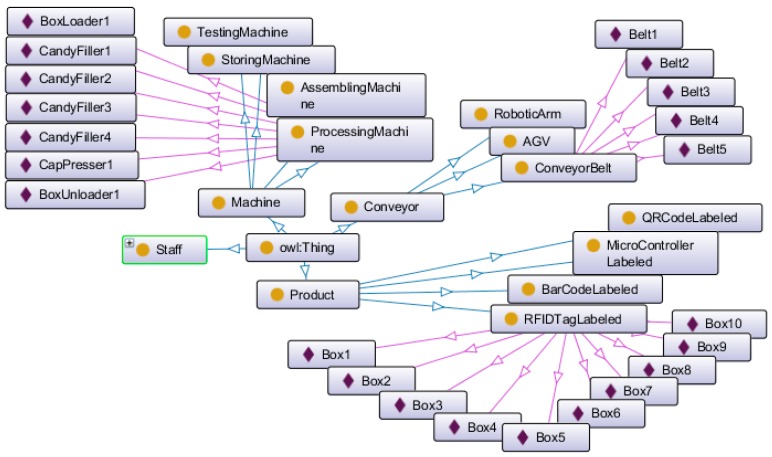
Ontology graph.

Both conveyor belts and machines are implemented as OPC UA servers. Ontologies (Figure 7) are built and deployed on the cloud along with data base. Note that, although three kinds of conveyors and four types of products are defined in Table 1, the UniqueCandy prototype only uses conveyor belts and RFID tag labeled products. As for machines, the candy fillers are processing machines, the box loader and the box unloader are storing machine, and the cap presser is assembling machine. The abovementioned items are disclosed in Figure 7. Apache Jena is also deployed on the cloud. A Bonding routine was developed using JAVA, it collects data from conveyor belts and machines and stores them into the database. It also invokes Jena with data read from the data base and the SWRL rules corresponding to application requirements.

With our previous work [11,12,13], the smart factory operates properly as designed. Different consumer orders can be processed by the UniqueCandy smart factory simultaneously. We use SWRL to describe rules for fault detecting and statistical analysis and the system behaves as designed.

## 5. Conclusions

In the implementation of smart factories, we apply several emerging technologies such as industrial Ethernet network, cloud computing, big data, and artificial intelligence to achieve the vertical integration of automation systems with information systems. Shop floor entities such as machines, conveyors, and products form a self-organized system based on intelligent negotiation, and the cloud assists self-organization based on industrial data analysis. In this way, we build a cloud-assisted and self-organized manufacturing system to combine the flexibility and fault tolerance of self-organized system and the global optimization abilities of the cloud. To address the real time processing requirements of increasing industrial data, we propose an integrated scheme of knowledge reasoning and semantic data. We successfully apply the cloud-assisted and self-organized framework and the knowledge reasoning and semantic data integration scheme to the benchmarking of a personalized candy packing application, for which the framework and the scheme are verified.

## Figures and Tables

**Table 1:** Object classification.

Main Classes	Sub Classes			
Machine	processing machine	testing machine	assembling machine	storing machine
Conveyor	conveyor belt	AGV	robotic arm that moves products	
Product	bar code labeled product	QR code labeled product	RFID tag labeled product	micro controller labeled product
Staff	operator	maintenance technician	team leader	manager

**Table 2:** Recommended language and related software tools for modeling.

Model	Language	Recommended Tool
information model	UML	JUDE-community
OPC UA model	OPC UA	UaModeler
ontology model	OWL 2.0	Protégé
application model	SWRL	Apache Jena
